# Unsaturated Fatty Acids Supplementation Reduces Blood Lead Level in Rats

**DOI:** 10.1155/2015/189190

**Published:** 2015-05-14

**Authors:** Anna Skoczyńska, Anna Wojakowska, Dorian Nowacki, Łukasz Bobak, Barbara Turczyn, Beata Smyk, Andrzej Szuba, Tadeusz Trziszka

**Affiliations:** ^1^Department of Internal and Occupational Diseases, Wroclaw Medical University, 50-556 Wroclaw, Poland; ^2^Department of Human Nutrition, Wroclaw University of Environmental and Life Sciences, 51-630 Wrocław, Poland; ^3^Department of Animal Products Technology and Quality Management, Wroclaw University of Environmental and Life Sciences, 51-630 Wrocław, Poland

## Abstract

Some dietary factors could inhibit lead toxicity. The aim of this study was to evaluate the effect of dietary compounds rich in unsaturated fatty acids (FA) on blood lead level, lipid metabolism, and vascular reactivity in rats. Serum metallothionein and organs' lead level were evaluated with the aim of assessing the possible mechanism of unsaturated FA impact on blood lead level. For three months, male Wistar rats that were receiving drinking water with (100 ppm Pb) or without lead acetate were supplemented *per os* daily with virgin olive oil or linseed oil (0.2 mL/kg b.w.) or egg derived lecithin fraction: “super lecithin” (50 g/kg b.w.). Mesenteric artery was stimulated *ex vivo* by norepinephrine (NE) administered at six different doses. Lecithin supplementation slightly reduced pressor responses of artery to NE. Lead administered to rats attenuated the beneficial effect of unsaturated FA on lipid metabolism and vascular reactivity to adrenergic stimulation. On the other hand, the super lecithin and linseed oil that were characterized by low omega-6 to omega-3 ratio (about 1) reduced the blood lead concentration. This effect was observed in lead poisoned rats (*p* < 0.0001) and also in rats nonpoisoned with lead (*p* < 0.05).

## 1. Introduction

Populations exposed to lead, the most ubiquitous xenobiotic in human environment, display increased cardiovascular morbidity and mortality [1, 2]. This applies to an exposure to lead in small doses that are known to induce arterial hypertension and lipid disorders, impact heart rate variability, and can induce peripheral arterial disease [3–6].

Epidemiological studies on the association between cardiovascular disease and the body lead burden, led since the 1970's, have showed a gradual decrease in blood lead levels in the world population [7]. It is a consequence of the withdrawal of lead from manufacturing processes as well as introduction of legal requirements and more widely understood principles of environmental protection on a global scale. It is also possible that some dietary factors, used in prevention of cardiovascular disease, inhibit lead cardiotoxicity. Lately, such effect has been attributed to omega-3 polyunsaturated fatty acids (PUFA): docosahexaenoic acid (DHA; 22 : 6) and eicosapentaenoic acid (EPA; 20 : 5) in relation to toxicity of another metal, that is, mercury [8, 9]. In our earlier study, we observed a decrease in blood cadmium level associated with increased urinary N-acetyl-*β*-glucosaminidase activity in hypercholesterolemic patients treated with evening primrose oil, rich in omega-6 fatty acids (FA) especially in *γ*-linolenic acid [10]. Furthermore, it was demonstrated that a higher maternal dietary intake of PUFA might limit lead toxicity in fetuses and infants during pregnancy and lactation [11]. The interaction between PUFA and lead affecting liver and serum fatty acid profiles was also shown in animal studies [12]. Fatty acids undergo interactions also with other metals; for example, they influence binding of cobalt to serum albumin in patients with fatty liver disease [13]. It is important as one of biomarkers for early diagnosis of myocardial ischemia is “ischemia-modified albumin,” measured by the albumin-cobalt-binding assay [14]. However, in general, there is little published data on the effect of natural unsaturated fatty acids on heavy metals' toxicity, especially in relation to lead.

On the other hand, many experimental studies very well documented that omega-3 PUFA, such as DHA and EPA, contained in marine algae, fatty fish, and fish oils, exert many positive effects on the circulatory system. In experimental models, a long-term consumption of lipid modified formula diet with DHA and EPA improved blood lipid pattern [15] and inflammatory status [16, 17] and also showed neuroprotective activity [18, 19]. Dietary fish oil augments endothelium-dependent vasorelaxation and this effect is associated with increased endothelial nitric oxide synthase (eNOS) expression and decreased oxidative stress [20, 21]. Additionally, DHA decreases arterial blood pressure in hypertensive rats [22]. All of these cardioprotective mechanisms of PUFA action are simultaneously the target for toxic effect of lead. There are therefore grounds to suspect that essential PUFA, such as DHA and EPA, could reduce the cardiotoxic effects of heavy metals.

The aim of this study was to evaluate the effect of dietary compounds rich in omega-3 and omega-6 fatty acids, of animal or vegetable origin, on blood lead level, lipid metabolism, and vascular reactivity to norepinephrine in rats. Since only exposure to low doses of lead is associated with increased cardiovascular risk, part of the study was performed using a model corresponding to the environmental exposure to lead. Apart from drinking water and chow, containing some amounts of lead, some rats did not receive any additional lead compounds. At the same time, the study was carried out on a group of rats poisoned with lead (corresponding to model of occupational exposure), in order to conduct in-depth analysis of the possible impact of PUFAs on the toxicological, metabolic, and functional effects of lead.

## 2. Materials and Methods

### 2.1. Sources of Unsaturated Fatty Acids

Two vegetable sources of unsaturated fatty acids: virgin olive oil (premium extravirgin olive oil, Angel Camacho Alimentacion S.L., Moron de la Frontera, Spain) and linseed oil (Vis Nature Linseed Oil, Aleksander Nowak, Wroclaw, Poland), were used. One animal source of omega-3 and omega-6 FA: egg yolk phospholipid fraction (super lecithin) obtained in Department of Animal Products Technology and Quality Management, Wroclaw University of Environmental and Life Sciences, from Lohmann Brown hens line [23], was also used.

Fatty acid content in the used ingredients was analyzed by gas chromatography. Samples (ca. 50 mg) of linseed oil, virgin olive oil, and super lecithin were dissolved in 4 mL of 0.5 M methanolic NaOH solution and heated under reflux for 2 min. After that 4 mL of BF_3_ (14% solution in methanol) was added and the mixtures were heated once again under reflux for 2 min. Solution after methylation was cooled and extracted with 6 mL of hexane. Hexane extracts were dried using anhydrous magnesium sulphate and evaporated under reduced pressure and residues dissolved in 1.5 mL of hexane. Prepared fatty acid methyl esters (FAME) were analyzed by gas chromatography (GC Agilent 6890N Series), using a 88% cyanopropyl, and 12% aryl polysiloxane column (HP-88, 100 m 0.25 mm 0.25 *μ*m) and MS detector (5973 MS Detector Agilent).

### 2.2. Animals

Male Wistar rats, 4–6 weeks of age and 200 ± 15 g bodyweight, were kept in the same room with natural lighting cycle, the stable temperature of 20°C, and the humidity of 55 ± 5%. Animals had free access to drinking water and to standard laboratory chow (Labofeed H; WPIK, Kcynia, Poland) and received care according to the criteria outlined in the* Guide for the Care and Use of Laboratory Animals *(National Research Council, 1996). This study was approved by the Local Ethics Committee of the Polish Academy of Science, no. 201061.

### 2.3. Treatment Protocol

Ninety three rats were randomly divided into eight groups: untreated rats (12 animals); virgin olive oil treated rats (olive oil, 12 animals); linseed oil treated rats (linseed oil, 12 animals); super lecithin treated rats (super lecithin, 12 animals); lead poisoned rats (Pb group, 10 animals); lead poisoned rats treated by virgin olive oil (Pb plus olive oil, 11 animals); lead poisoned rats treated by linseed oil (Pb plus linseed oil, 12 animals); lead poisoned rats treated by super lecithin (Pb plus super lecithin, 12 animals). For three months animals poisoned with lead were receiving drinking water with the addition of lead acetate at a final concentration of 100 ppm Pb (the solution was prepared daily), whereas rats nonpoisoned with lead were receiving standard drinking water. Simultaneously, depending on the group, rats were given virgin olive oil or linseed oil or super lecithin or were not given any fatty acids. Oils were given every day, orally, by pipette, at a volume of 0.2 mL/kg b.w. Super lecithin was given with chow, at a dose of 50 g/kg b.w. daily.

### 2.4. Reagents

All reagents were of analytical grade. All aqueous solutions were prepared with deionized water obtained by using ultrapure water system. All drugs concentrations were expressed in terms of freebase and prepared* ex tempore*. Used drugs are as follows: lead acetate (Fluka, Buchs, Switzerland), ketamine (Wholesale Veterinary Medicines Bayleg SJ, Ziemnice, Kunice, Poland), and norepinephrine HCl (Polfa SA, Warsaw, Poland),

### 2.5. Experiments* Ex Vivo*

After the three-month exposition to lead and/or supplementation with dietary unsaturated FA, experiments* ex vivo* were performed. Starting the night before the experiment, rats were fasted and the next day they were anesthetized intramuscularly with ketamine at a dose of 300 mg/kg. An isolated superior mesenteric artery preparation was obtained, immediately placed in the chamber of the Harvard Perfusion System and flushed with aerated modified Krebs solution of the following composition (in mM): NaCl 112.0, KCl 5.0, NaH_2_PO_4_ 1.0, MgCl_2_ 0.5, CaCl_2_2.5, NaHCO_3_ 25.0, D(+) glucose 11.2. The perfusate was aerated with a 95% O_2_ and 5% CO_2_ mixture and maintained at 30°C. The pH and osmolarity of the perfusate were 7.4 and 284 mOsm, respectively. Blood samples from the heart were collected, and animal was killed by cervical dislocation. An electronically controlled peristaltic pump was applied to perfuse mesenteric vessels at a constant flow rate of 8.4 mL/min. Changes in perfusion pressure (delta PP) were monitored with an APT 300 Pressure transducer and recorded on a TAM-D type 705/2 (Hugo Sachs Electronic, Germany). Norepinephrine (NE) at the dose of 0.01, 0.1, 0.5, 1.0, 3.0, and 5.0 *μ*g was dissolved in Krebs solution and injected into mesenteric superior artery at a volume of 100 *μ*L, every time after stabilization of the basal perfusion pressure. ED_50_NE (dose of NE inducing half of the maximal response) was estimated individually for each preparation.

### 2.6. Analytical Measurements

Lead levels in blood and mineralizated organs were determined by the atomic absorption spectrophotometer in an acetylate flame on a SOLAAR M6 (Thermo Elemental, Solaar House, Cambridge, UK). The accuracy and repeatability of the method were checked using control blood samples BCR produced by European Commission Joint Research Centre Institute for Reference Materials and Measurements and two level controls (ClinChec-Control I and II, Recipe Chemichals-Instruments, Munich, Germany) were used. Mineralization of samples obtained from kidney, liver, and heart was performed using High Performance Microwave Digestion System (Milestone, Ethos One, Sorisole, Italy, tube SK-12T). The serum total cholesterol, triglycerides, high density lipoprotein (HDL) cholesterol, HDL_2_, and HDL_3_ cholesterol levels were determined using enzymatic assay (SPINREACT, S.A. Ctra Santa Coloma, 7 E-17176 SantEsteve de Bas, Spain) by the Beckman DU 650 spectrophotometer (Beckman Instruments, Inc. 4300 Harbor Boulevard, Fullerton, CA California, US 92834). To measure precipitation of HDL_2_ and HDL_3 _cholesterol in serum, QUANTOLIP HDL (HDL_2_/HDL_3_) test (Technoclone GmbH, Vienna, Austria) was applied. Serum non-HDL cholesterol was calculated as a difference between total- and HDL-cholesterol concentrations. The serum cholesteryl ester transfer protein (CETP) and phospholipid transfer protein (PLTP) were determined using CETP Activity Assay Kit and PLTP Activity Assay Kit (BioVision Research Products, 2455-D Old Middlefield Way, Mountain View, CA 94043, USA) by the HITACHI F-2500 fluorescence spectrophotometer.

The concentration of serum metallothionein was determined using Rat MT ELISA Kit (Wuhan EIAab Science Co., Ltd., East Lake Hi-Tech Development Zone, Wuhan, China) by the BIOTEK-EPOCH 200–1000 spectrophotometer.

### 2.7. Statistical Analysis

Values are presented as a mean ± standard deviation and analyzed by analysis of variance (ANOVA/MANOVA) followed by* post hoc* comparison performed by Tukey RIR test. The strength of the linear association between variables was determined by the Pearson correlation coefficient (*r*).* Dose-response *curves graphs were approximated according to the equation: *y* = ΔPP_min⁡_ + (ΔPP_max⁡_ − ΔPP_min⁡_)/(1 + 10^∧^(lgD_50_NE − *x*)). Values of *p* less than 0.05 were considered significant. Statistical analysis was performed with STATISTICA 10.0 software.

## 3. Results

Fatty acid content in the used ingredients is presented in Table 1.

### 3.1. The Effect of Unsaturated Fatty Acids on Serum Lipids and Lipid Transfer Proteins

In groups of rats that drank water without added lead, in comparison to untreated animals, olive oil decreased serum non-HDL cholesterol (*p* < 0.01), and linseed oil decreased total cholesterol, mainly decreasing HDL_2 _cholesterol subclasse (*p* < 0.001), whereas super lecithin increased CETP activity (*p* < 0.01) and increased HDL_3_ cholesterol. In groups of rats that drank water contaminated with lead, olive oil and linseed oil did not significantly impact serum lipids in comparison to Pb group. Supplementation with super lecithin caused increase in non-HDL cholesterol (*p* < 0.01) that was associated with increase in CETP and decrease in PLTP activities (Table 2).

Multiple regression analysis showed the existence of the linear correlation between CETP and total cholesterol (*r* = 0.28066; *p* < 0.01), a weaker correlation between CETP and non-HDL cholesterol (*r* = 0.22664; *p* < 0.05), and linear correlations between PLTP and TG (*r* = 0.46887; *p* < 0.001), HDL_2_ cholesterol (*r* = 0.40918; *p* < 0.001), and HDL_3_ cholesterol (*r* = −0.4569; *p* < 0.001).

### 3.2. The Reactivity of Isolated Mesenteric Artery to Norepinephrine (NE)

**Figure 1:**
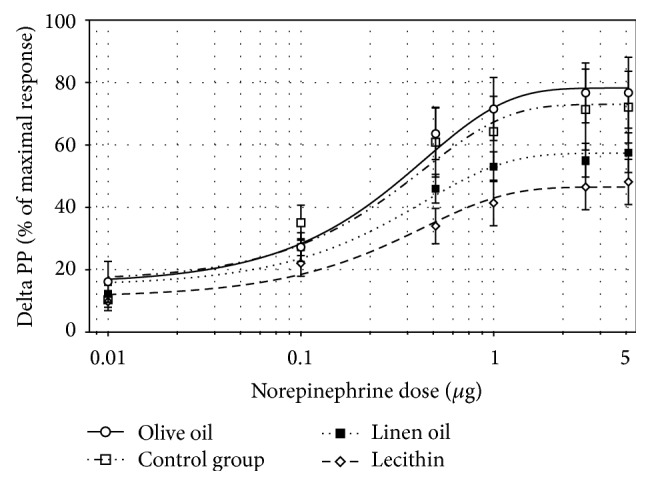
Dose-response curve to norepinephrine in rats treated with olive oil, or linseed oil, or super lecithin or not treated with any fatty acids.

**Figure 2:**
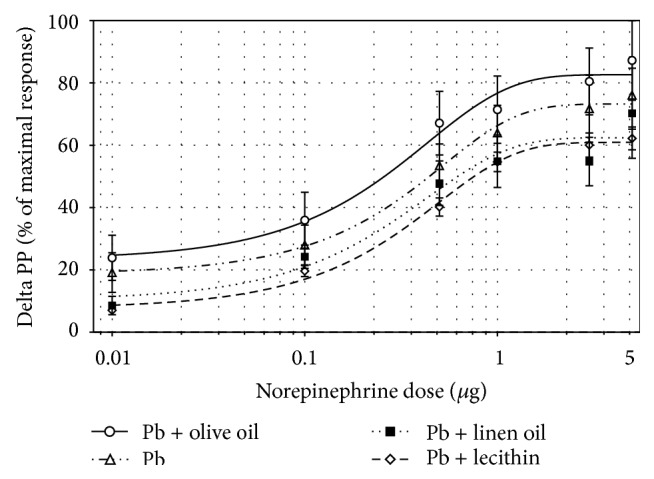
Dose-response curve to norepinephrine in lead poisoned rats treated with olive oil, or linseed oil, or super lecithin or not treated with any fatty acids.

In rats which drank water without lead, super lecithin slightly reduced pressor responses to NE injected at doses from 0.1 to 5.0 *μ*g and increased D_50_NE (*p* < 0.01); at the same time, linseed oil increased D_50_NE (*p* < 0.05) in comparison to animals which were not treated with fatty acids. In rats which drank water contaminated with lead, unsaturated fatty acids did not influence significantly the vascular reactivity to NE (Table 3; Figures 1 and 2).

### 3.3. The Effect of Unsaturated Fatty Acids on the Blood Lead Level (Pb-B) and Serum Metallothionein

In rats nonpoisoned with lead, two sources of FA: linseed oil and super lecithin, decreased the Pb-B (*p* < 0.05), whereas the effect of virgin olive oil was not significant (*p* = 0.0643). In rats poisoned with lead and supplemented with linseed oil or super lecithin, Pb-B was lower (*p* = 0.00000 and *p* = 0.00004, resp.) in comparison to Pb-B measured in animals exposed to lead acetate without supplementation with FA. Using olive oil did not lead to significant decrease in Pb-B (*p* = 0.229).

In rats not poisoned with lead, the decrease in Pb-B induced by linseed oil (but not by super lecithin) was associated with the increase in serum MT concentration (Table 4).

In rats treated with lead and super lecithin, lead concentration in the liver was greater (*p* < 0.001) than in remaining groups. Simultaneously, rats treated with lead and super lecithin displayed a lower level of lead in the kidney than rats given only lead (*p* < 0.05).

Rats treated with lead and virgin olive oil also had lower, in comparison to Pb rats, lead concentration in the kidney (*p* < 0.01), but they displayed higher (*p* < 0.001) lead level in the heart (Table 4).

Multivariate analysis of variance showed an existence of interaction between exposure to lead and treatment with unsaturated FA in their influence on Pb-B: F (1; 89) = 7.2023; *p* = 0.008. This was the only interaction between lead and unsaturated FA in their effects on the measured toxicological, metabolic, and functional parameters.

## 4. Discussion

In various experimental models, long-term consumption of lipid modified formula diet with DHA and EPA decreased non-HDL cholesterol and impacted HDL [15, 24]. Also in this study, a three-month dietary supplementation with unsaturated FA such as omega-3 and omega-6 FA, contained in vegetable (virgin olive oil or linseed oil) or animal (super lecithin) sources, influenced blood lipid pattern in healthy rats. Virgin olive oil decreased non-HDL cholesterol, and linseed oil reduced total and HDL_2 _cholesterol, whereas super lecithin increased HDL_3_ cholesterol level and CETP activity.

On the contrary, a three-month dietary supplementation with olive oil or linseed oil in rats poisoned with lead did not significantly affect serum lipids or lipid transfer proteins. The relationship between blood lead level and lipid metabolism, studied in many experimental models, is described in a very varied, sometimes contradictory manner, from proatherogenic action of lead on lipid metabolism to a lack of connection between this metal and lipids [25–27]. It certainly depends on the kind of experiment and used doses of lead. Additionally, this study suggests that the relationship between blood lead and lipids' levels could strictly depend on the content of unsaturated FA in the diet. This applies mainly to super lecithin, egg yolk phospholipid fraction obtained from Lohmann Brown hens' line [23]. Poisoning with lead together with dietary supplementation with super lecithin caused an increase in non-HDL cholesterol; this effect was different from the effect of exposure to lead acetate with no supplementation (decrease in non-HDL-C) and was different from the effect of super lecithin administered without lead (non-HDL-C without change).

Simultaneously, the effect of super lecithin on non-HDL-C in rats was related to some increase in activity of cholesteryl ester transfer protein. Lecithin-induced CETP stimulation may explain the lecithin-induced increase in non-HDL cholesterol in lead-poisoned rats, as an existence of a positive correlation between CETP and non-HDL cholesterol was shown [28]. Such correlation was shown also in our study. Similarly, the effect of some PUFAs on rats' HDL_2_ cholesterol seems to be a consequence of PUFAs' weak inhibition of PLTP activity, since a linear dependence between PLTP and HDL_2_ cholesterol was shown in this experiment, as well as in other studies [29].

Obtained results indicate also that the effect of unsaturated fatty acids on lipid metabolism in rats poisoned with lead is less beneficial or even adverse, in comparison to one observed in nonpoisoned animals. For example, in rats treated with lead, neither non-HDL cholesterol reduction by virgin olive oil, nor decrease in total cholesterol induced by linseed oil, nor increase in the HDL_3_ cholesterol induced by super lecithin was observed, in contrast to the effect of unsaturated FA in nonpoisoned rats. In rats poisoned with lead, and lecithin induced increase in nonHDL cholesterol, which can be recognized as an adverse effect of the FA supplementation (Table 2).

Other studies have shown that the beneficial effect of omega-3 FA in healthy rats was associated with PUFAs' inhibitory impact on cardiac [30] and vascular adrenergic response [31]. Likewise, in this study, super lecithin (and linseed oil) attenuated vasopressor response to exogenous norepinephrine in healthy rats (Figure 1). These results correspond also to an observation of a reduced vasoconstriction in response to NE in mice supplemented for a long time with dietary omega-3 FA [32, 33].

Recently, it has also been described that DHA can inhibit gene expression of cyclooxygenase-2, decreasing tension and causing an easier relaxation of rat's aorta [24] and directly decreasing blood pressure by activating BK channels in vascular smooth muscle [34]. On the other hand, Engler et al. observed that DHA did not influence vascular reactivity to NE in SHR rats [22]. In our study, the vasoconstriction in response to NE in lead-exposed rats was unchanged by unsaturated fatty acids supplementation (Figure 2). Thus, it can be concluded that lead poisoning, as in case of lipid metabolism, attenuated the vascular effects of omega-3 FA contained in super lecithin and linseed oil.

The most important observation in this study, as it has been noted probably for the first time, is a significant reduction of blood lead concentration, induced by ingredients rich in omega-3 and omega-6 fatty acids, such as super lecithin and linseed oil. This effect was observed in lead-poisoned rats, as well as in nonpoisoned animals. The effect of virgin olive oil on Pb-B was much weaker (not statistically significant). Of course, the difference between actions of various preparations could be related to various contents of lead and other toxic or essential metals and to interactions between them on the level of intestinal absorption, intra- and extracellular transport, and mechanisms of elimination. However, the difference could also be related to various omega-6 to omega-3 fatty acid ratio. This ratio was 1,45 in super lecithin, 0,45 in linseed oil, and 7,71 in olive oil (Table 1). The optimal ratio of omega-6 to omega-3 FA is estimated to be approximately 1. A low omega-6/omega-3 FA ratio is more desirable in reducing the risk of many chronic diseases, including cardiovascular pathology [35]. In our study it could be responsible for beneficial changes in vascular reactivity in nonpoisoned rats. It should be asked, what may be a link between a low omega-6/omega-3 FA ratio and decreased blood lead level. Recently, it has been shown that omega-3 FA such as EPA and DHA are sources of specialized proresolving mediators (SPM), including E-series and D-series resolvins, that possess potent anti-inflammatory and proresolving actions. SPM, among other, stimulate macrophage uptake and macrophage-directed apoptotic cells' clearance mechanisms [17]. In rats, exogenous lead is located mainly within hepatocytes [36], renal tubulointerstitium [37], type II pneumocytes, and macrophages [38]. It is possible that SPM, produced in higher quantities in rats given super lecithin than in rats given other FA sources or water only, enhanced lead uptake, for example, by hepatic macrophages. To such a SPM action it could be attributed that the liver highest lead concentration was observed, in this study, in rats poisoned with lead and treated with super lecithin (3–5 times higher than in other poisoned rats).

It is quite possible that super lecithin enhanced also biliary secretion of lead. Super lecithin increased lead concentration in the liver, as well as CETP activity in blood, which was associated with a slightly reduced PLTP activity. Most long-chain polyunsaturated fatty acids cause an increase in bile production and secretion in the liver [39] which occur together with an increase in serum cholesterol and CETP activity [40] and decrease in PLTP activity [41]. In contrast to super lecithin effect, the decrease in Pb-B induced by linseed oil was associated with an increase in serum MT concentration (Table 4), indicating an increased turnover of lead in rat organism.

There are more questions, such as whether the lead reduction in blood results from reduced absorption of this metal in the gastrointestinal tract, chelating action of omega-3 and omega-6 FA, or increased accumulation of lead in tissues. In rats, lead cumulates mainly in kidneys, liver, lungs, spleen, and cardiac muscle (as an intermediately fast exchangeable pool) and in bone (as a slowly exchangeable pool). In our study, distribution of lead (maximal in kidneys and minimal in the heart) was typical for a compartment that is characterized by a slower turnover rate and transit time, that is, for intracellular space (soft tissues and erythrocytes) [42]. We suppose that unsaturated fatty acids including PUFAs could increase urinary elimination of lead, since a reduced concentration of lead in kidney was shown in all rats nonpoisoned with lead and treated with FA, as well as in rats poisoned with lead and treated with olive oil or super lecithin.

In summary, in rats nonpoisoned with lead, the mean blood lead level was lower than 100 *μ*g/L, which means it corresponded to the environmental exposure to this metal [7, 43–45]. Probably, it reflected the presence of lead in drinking water and in chow. Unsaturated fatty acids such as omega-3 and omega-6 fatty acids exerted the beneficial effect on lipid metabolism and vascular reactivity in these rats. Lead acetate that was administered to poisoned rats in drinking water for three months caused the increase in mean blood lead level to 679 *μ*g/L, which corresponded to the occupational exposure [45, 46]. Poisoning with lead attenuated the beneficial effect of unsaturated fatty acids on lipid metabolism and vascular reactivity to adrenergic stimulation. On the other hand, the super lecithin and linseed oil that were characterized by a low omega-6 to omega-3 ratio (about 1) reduced blood lead concentration. This effect was shown in rats receiving lead in drinking water, but also, although being to a smaller degree, in rats nonpoisoned with lead. It is probable that the lowering of blood lead level by omega-3 fatty acids resulted, at least in part, from enhanced hepatic uptake of lead or chelating action of fatty acids. The diversity and inconsistency of observations concerning lead effect on cardiovascular system, as described by different authors in literature, could derive not only from various models of lead poisoning, but also from various dietary FAs contents.

## Figures and Tables

**Table 1:** Fatty acid content in the used ingredients.

Fatty acid profile	Olive oil (%)	Linseed oil (%)	Super lecithin (%)
C_14:0_	ND	ND	0.40
C_14:1_	ND	ND	0.16
C_16:0_	17.82	8.18	26.36
C_16:1_	1.23	ND	2.52
C_17:0_	0.16	ND	0.24
C_18:0_	6.47	7.54	14.05
C_18:1_	63.96	27.15	29.66
C_18:2_	8.43	17.16	13.08
C_18:3_	1.19	39.52	3.12
C_20:2_	0.23	0.29	0.18
C_20:4_	0.51	0.16	2.41
C_20:5_	ND	ND	0.58
C_22:6_	ND	ND	7.12
*ω*-3	1.19	39.52	10.82
*ω*-6	9.17	17.61	15.67
*ω*-6/*ω*-3	7.71	0.45	1.45
Saturated FA	24.45	15.72	41.05
Unsaturated FA	75.55	84.28	58.83
MUFA	65.19	27.15	32.34
PUFA	10.36	57.13	26.49

MUFA = monounsaturated fatty acids.

ND = not detected.

**Table 2:** Influence of dietary supplementation with olive oil or linseed oil or super lecithin on serum lipids and lipid transfer proteins in nonpoisoned rats and rats poisoned with lead.

Group of rats	Lipids						Proteins	
	Chol mmol/L	TG mmol/L	HDL-C mmol/L	HDL_3_-C mmol/L	HDL_2_-C mmol/L	Non HDL-C mmol/L	PLTP pmol/*µ*l/hr	CETP pmol/*µ*l/hr
Untreated (*n* = 12)	1.0 ± 0.23	1.04 ± 0.51	0.88 ± 0.23	0.45 ± 0.16	0.45 ± 0.07	0.62 ± 0.14	70.2 ± 23.1	28.7 ± 3.9
Olive oil (*n* = 12)	1.35 ± 0.25	1.02 ± 0.3	0.95 ± 0.21	0.50 ± 0.12	0.45 ± 0.18	0.4 ± 0.14^b^	82.3 ± 18.9	27.9 ± 5.1
Linseed oil (*n* = 12)	1.32 ± 0.15^c^	0.81 ± 0.29	0.8 ± 0.09	0.52 ± 0.07	0.28 ± 0.07^a^	0.53 ± 0.1	61.7 ± 10.9	31.8 ± 5.6
Super lecithin (*n* = 12)	1.57 ± 0.19	0.95 ± 0.25	0.95 ± 0.15	0.56 ± 0.10^c^	0.39 ± 0.10	0.62 ± 0.21	61.4 ± 11.2	34.4 ± 3.6^b^

Pb (*n* = 10)	1.43 ± 0.16	0.99 ± 0.64	0.93 ± 0.11	0.51 ± 0.06	0.42 ± 0.08	0.50 ± 0.12	74.3 ± 17.7	29.7 ± 8.3
Pb + olive oil (*n* = 11)	1.42 ± 0.15	1.11 ± 0.46	0.88 ± 0.25	0.44 ± 0.14	0.44 ± 0.16	0.54 ± 0.2	85.4 ± 14.2	28.7 ± 2.6
Pb + linseed oil (*n* = 12)	1.33 ± 0.12	0.81 ± 0.18	0.87 ± 0.13	0.51 ± 0.07	0.33 ± 0.13	0.42 ± 0.18	57.5 ± 13.6	27.9 ± 6.7
Pb + super lecithin (*n* = 12)	1.62 ± 0.26	1.11 ± 0.20	0.91 ± 0.17	0.57 ± 0.10	0.34 ± 0.10	0.70 ± 0.2^y^	60.1 ± 6.8^z^	34.1 ± 2.2

Results are presented as mean ± SD. Statistically significant differences: ^a^ *p* < 0.001, ^b^ *p* < 0.01; ^c^ *p* < 0.05 in comparison to untreated rats; ^y^ *p* < 0.01, ^z^ *p* < 0.05 in comparison to group of rats given lead acetate (Pb group).

Chol: cholesterol; TG: triglycerides; HDL-C: high density cholesterol; HDL_3_-C: HDL_3_ cholesterol; HDL_2_-C: HDL_2_ cholesterol; non HDL-C: non HDL cholesterol; PLTP: phospholipid transfer protein; CETP: cholesteryl ester transfer protein.

**Table 3:** Influence of virgin olive oil, linseed oil, and super lecithin (lecithin) on changes of perfusion pressure (in mmHg) induced by NE, and on the D_50_NE.

Group	Changes of perfusion pressure (in mmHg) in response to NE at dose of:						D_50_NE (*μ*g)
	0.01 *μ*g	0.1 *μ*g	0.5 *μ*g	1.0 *μ*g	3.0 *μ*g	5.0 *μ*g	
Control (*n* = 12)	13.0 ± 10.3	44.0 ± 24.4	76.3 ± 48.5	80.5 ± 49.2	89.5 ± 56.3	90.4 ± 49.8	0.20 ± 0.18
Olive oil (*n* = 12)	20.3 ± 27.0	34.2 ± 19.9	79.7 ± 35.8	89.7 ± 43.9	96.2 ± 41.7	96.3 ± 49.3	0.44 ± 0.25^∗^
Linseed oil (*n* = 12)	15.4 ± 11.8	34.2 ± 11.8	57.7 ± 19.9	66.5 ± 20.7	69.1 ± 23.3	72.1 ± 27.7	0.31 ± 0.29^∗^
Lecithin (*n* = 12)	12.2 ± 12.5	27.7 ± 18.0^∗^	42.6 ± 24.6^∗^	51.9 ± 31.7^∗^	58.3 ± 31.5^∗^	60.4 ± 31.5^∗^	0.48 ± 0.31^∗∗^

Pb (*n* = 10)	24.0 ± 25.3	35.1 ± 25.4	67.0 ± 27.4	80.2 ± 34.9	89.9 ± 42.7	95.4 ± 43.2	0.34 ± 0.21
Pb + olive oil (*n* = 11)	30.0 ± 30.2	45.1 ± 37.3	84.1 ± 42.4	89.5 ± 44.9	100.9 ± 44.5	109.4 ± 53.0	0.28 ± 0.21
Pb + linseed oil (*n* = 12)	10.7 ± 12.8	30.4 ± 16.3	59.9 ± 30.9	68.8 ± 36.7	68.7 ± 33.8	88.1 ± 62.8	0.32 ± 0.21
Pb + lecithin (*n* = 12)	8.83 ± 3.71	24.7 ± 7.61	50.4 ± 12.7	68.5 ± 13.5	75.3 ± 16.8	78.0 ± 15.9	0.40 ± 0.18

Results are presented as mean ± SD. Differences statistically significant: ^∗∗^ *p* < 0.01; ^∗^ *p* < 0.05 in comparison to untreated rats.

**Table 4:** Lead and metallothionein (MT) concentrations in tissues of rats poisoned with lead and/or treated with PUFA.

Group of rats	Lead concentration				MT
	Blood (*μ*g/L)	Liver (mg/g)	Kidney (mg/g)	Heart (mg/g)	Serum (ng/mL)
Untreated (*n* = 10)	78.6 ± 93.1	0.048 ± 0.061	1.09 ± 2.02	0.016 ± 0.022	6.55 ± 1.63
Olive oil (*n* = 12)	34.9 ± 22.8	0.079 ± 0.107	0.459 ± 0.55	0.074 ± 0.068	7.23 ± 2.44
Linseed oil (*n* = 11)	5.94 ± 5.56^c^	0.038 ± 0.076	0.281 ± 0.69	0.019 ± 0.018	8.46 ± 2.82^a^
Super lecithin (*n* = 12)	4.76 ± 4.53^c^	0.034 ± 0.036	0.175 ± 0.19	0.045 ± 0.061	7.16 ± 1.74

Pb (*n* = 10)	678.7 ± 429.2	0.251 ± 0.312	46.01 ± 38.63	0.101 ± 0.122	6.56 ± 0.47
Pb + olive oil (*n* = 11)	582.4 ± 284.9	0.192 ± 0.275	18.72 ± 18.70^y^	0.233 ± 0.172^x^	7.18 ± 0.77
Pb + linseed oil (*n* = 10)	214.8 ± 73.0^x^	0.342 ± 0.252	46.66 ± 29.6	0.032 ± 0.035	9.17 ± 4.31^x^
Pb + lecithin (*n* = 12)	288.8 ± 51.4^x^	1.135 ± 1.08^x^	26.34 ± 15.4^z^	0.089 ± 0.041	7.33 ± 2.12

Results are presented as means ± SD. Statistically significant differences: ^a^ *p* < 0.001; ^c^ *p* < 0.05; in comparison to untreated rats; ^x^ *p* < 0.001; ^y^ *p* < 0.01; ^z^ *p* < 0.05 in comparison to group of rats given lead acetate (Pb group).

## References

[1] Lustberg M., Silbergeld E. (2002). Blood lead levels and mortality. *Archives of Internal Medicine*.

[2] Weisskopf M. G., Jain N., Nie H. (2009). A prospective study of bone lead concentration and death from all causes, cardiovascular diseases, and cancer inthe Department of Veterans Affairs Normative Aging Study. *Circulation*.

[3] Park S. K., O'Neill M. S., Vokonas P. S. (2008). Air pollution and heart rate variability: effect modification by chronic lead exposure. *Epidemiology*.

[4] Park S. K., Mukherjee B., Xia X. (2009). Bone lead level prediction models and their application to examine the relationship of lead exposure and hypertension in the third national health and nutrition examination survey. *Journal of Occupational and Environmental Medicine*.

[5] Kasperczyk S., Birkner E., Kasperczyk A., Kasperczyk J. (2005). Lipids, lipid peroxidation and 7-ketocholesterol in workers exposed to lead. *Human and Experimental Toxicology*.

[6] Navas-Acien A., Selvin E., Sharrett A. R., Calderon-Aranda E., Silbergeld E., Guallar E. (2004). Lead, cadmium, smoking, and increased risk of peripheral arterial disease. *Circulation*.

[7] Menke A., Muntner P., Batuman V., Silbergeld E. K., Guallar E. (2006). Blood lead below 0.48 *μ*mol/L (10 *μ*g/dL) and mortality among US adults. *Circulation*.

[8] Wennberg M., Strömberg U., Bergdahl I. A. (2012). Myocardial infarction in relation to mercury and fatty acids from fish: a risk-benefit analysis based on pooled Finnish and Swedish data in men. *American Journal of Clinical Nutrition*.

[9] Houston M. C. (2011). Role of mercury toxicity in hypertension, cardiovascular disease, and stroke. *Journal of Clinical Hypertension*.

[10] Skoczyńska A., Smolik R. (1995). Effect of gamma-linolenic acid on cardiovascular risk factors on the basis of clinical studies with Oeparol. *Materials of Symposium on Oil from the Seeds of Evening Primrose Oil in the Prevention and Therapy*.

[11] Arora M., Ettinger A. S., Peterson K. E. (2008). Maternal dietary intake of polyunsaturated fatty acids modifies the relationship between lead levels in bone and breast milk. *Journal of Nutrition*.

[12] Knowles S. O., Donaldson W. E. (1990). Dietary modification of lead toxicity: effects on fatty acid and eicosanoid metabolism in chicks. *Comparative Biochemistry and Physiology Part C: Comparative Pharmacology*.

[13] Amirtharaj G. J., Natarajan S. K., Mukhopadhya A. (2008). Fatty acids influence binding of cobalt to serum albumin in patients with fatty liver. *Biochimica et Biophysica Acta—Molecular Basis of Disease*.

[14] Lu J., Stewart A. J., Sadler P. J., Pinheiro T. J. T., Blindauer C. A. (2012). Allosteric inhibition of cobalt binding to albumin by fatty acids: implications for the detection of myocardial ischemia. *Journal of Medicinal Chemistry*.

[15] Ukropec J., Reseland J. E., Gasperikova D. (2003). The hypotriglyceridemic effect of dietary n-3 FA is associated with increased *β*-oxidation and reduced leptin expression. *Lipids*.

[16] Figueras M., Olivan M., Busquets S., López-Soriano F. J., Argilés J. M. (2011). Effects of eicosapentaenoic acid (EPA) treatment on insulin sensitivity in an animal model of diabetes: improvement of the inflammatory status. *Obesity*.

[17] Serhan C. N., Chiang N. (2013). Resolution phase lipid mediators of inflammation: agonists of resolution. *Current Opinion in Pharmacology*.

[18] Belayev L., Khoutorova L., Atkins K. D., Bazan N. G. (2009). Robust docosahexaenoic acid-mediated neuroprotection in a rat model of transient, focal cerebral ischemia. *Stroke*.

[19] Mills J. D., Hadley K., Bailes J. E. (2011). Dietary supplementation with the Omega-3 fatty acid docosahexaenoic acid in traumatic brain injury. *Neurosurgery*.

[20] López D., Orta X., Casós K. (2004). Upregulation of endothelial nitric oxide synthase in rat aorta after ingestion of fish oil-rich diet. *American Journal of Physiology: Heart and Circulatory Physiology*.

[21] Nyby M. D., Matsumoto K., Yamamoto K. (2005). Dietary fish oil prevents vascular dysfunction and oxidative stress in hyperinsulinemic rats. *The American Journal of Hypertension*.

[22] Engler M. M., Engler M. B., Pierson D. M., Molteni L. B., Molteni A. (2003). Effects of docosahexaenoic acid on vascular pathology and reactivity in hypertension. *Experimental Biology and Medicine*.

[23] Noszczyk-Nowak A., Pasławska U., Nicpoń J. (2013). Influence of docosahexaenoic acid obtained from new generation of eggs on the repolarisation of ventricles in pigs with experimental tachycardiomyopathy. *Bulletin of the Veterinary Institute in Pulawy*.

[24] Chen J., Jiang Y., Liang Y. (2012). DPA n-3, DPA n-6 and DHA improve lipoprotein profiles and aortic function in hamsters fed a high cholesterol diet. *Atherosclerosis*.

[25] Kojima M., Masui T., Nemoto K., Degawa M. (2004). Lead nitrate-induced development of hypercholesterolemia in rats: sterol-independent gene regulation of hepatic enzymes responsible for cholesterol homeostasis. *Toxicology Letters*.

[26] Palacios J., Roman D., Cifuentes F. (2012). Exposure to low level of arsenic and lead in drinking water from Antofagasta city induces gender differences in glucose homeostasis in rats. *Biological Trace Element Research*.

[27] Sharma S., Raghuvanshi B. P., Shukla S. (2014). Toxic effects of lead exposure in rats: involvement of oxidative stress, genotoxic effect, and the beneficial role of N-acetylcysteine supplemented with selenium. *Journal of Environmental Pathology, Toxicology and Oncology*.

[28] Barter P. J., Brewer H. B., Chapman M. J., Hennekens C. H., Rader D. J., Tall A. R. (2003). Cholesteryl ester transfer protein: a novel target for raising HDL and inhibiting atherosclerosis. *Arteriosclerosis, Thrombosis, and Vascular Biology*.

[29] C. Cheung M., Wolfbauer G., Brown B. G., J. Albers J. (1999). Relationship between plasma phospholipid transfer protein activity and HDL subclasses among patients with low HDL and cardiovascular disease. *Atherosclerosis*.

[30] Brochot A., Guinot M., Auchere D. (2009). Effects of alpha-linolenic acid vs. docosahexaenoic acid supply on the distribution of fatty acids among the rat cardiac subcellular membranes after a short- or long-term dietary exposure. *Nutrition & Metabolism*.

[31] Zeydanli E. N., Turan B. (2009). Omega-3E treatment regulates matrix metalloproteinases and prevents vascular reactivity alterations in diabetic rat aorta. *Canadian Journal of Physiology and Pharmacology*.

[32] Mustad V. A., DeMichele S., Huang Y.-S. (2006). Differential effects of n-3 polyunsaturated fatty acids on metabolic control and vascular reactivity in the type 2 diabetic *ob/ob* mouse. *Metabolism: Clinical and Experimental*.

[33] Lamping K. G., Nuno D. W., Coppey L. J. (2013). Modification of high saturated fat diet with n-3 polyunsaturated fat improves glucose intolerance and vascular dysfunction. *Diabetes, Obesity and Metabolism*.

[34] Hoshi T., Wissuwa B., Tian Y. (2013). Omega-3 fatty acids lower blood pressure by directly activating large-conductance Ca^2+^-dependent K^+^ channels. *Proceedings of the National Academy of Sciences of the United States of America*.

[35] Simopoulos A. P. (2002). The importance of the ratio of omega-6/omega-3 essential fatty acids. *Biomedicine and Pharmacotherapy*.

[36] Jarrar B. M., Taib N. T. (2012). Histological and histochemical alterations in the liver induced by lead chronic toxicity. *Saudi Journal of Biological Sciences*.

[37] Rodríguez-Iturbe B., Sindhu R. K., Quiroz Y., Vaziri N. D. (2005). Chronic exposure to low doses of lead results in renal infiltration of immune cells, NF-kappaB activation, and overexpression of tubulointerstitial angiotensin II. *Antioxidants and Redox Signaling*.

[38] Kaczyńska K., Walski M., Szereda-Przestaszewska M. (2013). Long-term ultrastructural indices of lead intoxication in pulmonary tissue of the rat. *Microscopy and Microanalysis*.

[39] Werner A., Havinga R., Bos T., Bloks V. W., Kuipers F., Verkade H. J. (2005). Essential fatty acid deficiency in mice is associated with hepatic steatosis and secretion of large VLDL particles. *The American Journal of Physiology—Gastrointestinal and Liver Physiology*.

[40] Gautier T., Masson D., Pais de Barros J. P. (2000). Human apolipoprotein C-I accounts for the ability of plasma high density lipoproteins to inhibit the cholesteryl ester transfer protein activity. *The Journal of Biological Chemistry*.

[41] de Faria E. C., Gebrin A. C., Júnior W. N., Castilho L. N. (2004). Phospholipid transfer protein activity in two cholestatic patients. *Sao Paulo Medical Journal*.

[42] Castellino N., Aloj S. (1969). Intracellular distribution of lead in the liver and kidney of the rat. *British Journal of Industrial Medicine*.

[43] Navas-Acien A., Guallar E., Silbergeld E. K., Rothenberg S. J. (2007). Lead exposure and cardiovascular disease—a systematic review. *Environmental Health Perspectives*.

[44] Ekong E. B., Jaar B. G., Weaver V. M. (2006). Lead-related nephrotoxicity: a review of the epidemiologic evidence. *Kidney International*.

[45] Shih R. A., Hu H., Weisskopf M. G., Schwartz B. S. (2007). Cumulative lead dose and cognitive function in adults: a review of studies that measured both blood lead and bone lead. *Environmental Health Perspectives*.

[46] Cooper W. C., Wong O., Kheifets L. (1985). Mortality among employees of lead battery plants and lead-producing plants, 1947–1980. *Scandinavian Journal of Work, Environment and Health*.

